# Potential evidence for epigenetic biomarkers of metabolic syndrome in human whole blood in Latinos

**DOI:** 10.1371/journal.pone.0259449

**Published:** 2021-10-29

**Authors:** Keane Urashima, Anastasia Miramontes, Luis A. Garcia, Dawn K. Coletta

**Affiliations:** 1 Department of Physiology, University of Arizona, Tucson, Arizona, United States of America; 2 Department of Medicine, Division of Endocrinology, University of Arizona, Tucson, Arizona, United States of America; 3 Center for Disparities in Diabetes Obesity, and Metabolism, University of Arizona, Tucson, Arizona, United States of America; University of Oslo, NORWAY

## Abstract

Metabolic syndrome (MetS) is highly prevalent worldwide. In the United States, estimates show that more than 30% of the adult population has MetS. MetS consists of multiple phenotypes, including obesity, dyslipidemia, and impaired glucose tolerance. Therefore, identifying the molecular mechanisms to explain this complex disease is critical for diagnosing and treating MetS. We previously showed 70 increased genes and 20 decreased genes in whole blood in MetS participants. The present study aimed to identify blood-based DNA methylation biomarkers in non-MetS *versus* MetS participants. The present study analyzed whole blood DNA samples from 184 adult participants of Latino descent from the Arizona Insulin Resistance (AIR) registry. We used the National Cholesterol Education Program Adult Treatment Panel III (NCEP: ATP III) criteria to identify non-MetS (n = 110) and MetS (n = 74) participants. We performed whole blood methylation analysis on select genes: ATP Synthase, H+ Transporting mitochondrial F1 Complex, Epsilon Subunit (*ATP5E)*, Cytochrome C Oxidase Subunit VIc (*COX6C)*, and Ribosomal Protein L9 (*RPL9)*. The pyrosequencing analysis was a targeted approach focusing on the promoter region of each gene that specifically captured CpG methylation sites. In MetS participants, we showed decreased methylation in two CpG sites in *COX6C* and three CpG sites in *RPL9*, all p < 0.05 using the Mann-Whitney U test. There were no *ATP5E* CpG sites differently methylated in the MetS participants. Furthermore, while adjusting for age, gender, and smoking status, logistic regression analysis reaffirmed the associations between MetS and mean methylation within *COX6C* and *RPL9* (both p < 0.05). In addition, Spearman’s correlation revealed a significant inverse relationship between the previously published gene expression data and methylation data for *RPL9* (p < 0.05). In summary, these results highlight potential blood DNA methylation biomarkers for the MetS phenotype. However, future validation studies are warranted to strengthen our findings.

## Introduction

Metabolic syndrome (MetS) is a collection of metabolic phenotypes associated with diseases such as obesity, type 2 diabetes mellitus, dyslipidemia, and cardiovascular disease [1–3]. Classification of the MetS is based on the following National Cholesterol Education Program Adult Treatment Panel III (NCEP: ATP III) criteria: waist circumference (>102 cm men / >88 cm women), fasting plasma glucose (≥110 mg/dL), triglycerides (≥150 mg/dL), high-density lipoprotein cholesterol (<40mg/dL men / <50 mg/dL women) and high blood pressure (≥130/≥80 mmHg) [4]. In recent decades, it has become increasingly important to understand the molecular mechanisms of MetS because over a third of all United States adults met the NCEP: ATP III criteria [5].

The pathophysiological mechanisms of MetS are complex and are not entirely known. There have been many proposed physiological mechanisms, including insulin resistance, neurohormonal activation, and chronic low-grade inflammation [6]. Early diagnosis of the MetS is critical so that physicians can intervene and manage the disease to delay the progression of these physiological mechanisms. Identifying non-invasive blood-based biomarkers for MetS may aid in diagnosing and screening those patients at risk of developing MetS. Importantly, these biomarkers may help in the management and treatment of patients with this disease.

A systemic review by Srikanthan *et al*. highlighted several inflammatory and anti-inflammatory blood biomarkers for MetS [7]. In addition, a review by O’Neill *et al*. described novel blood-based biomarkers for MetS, including DNA genetic variants, microRNA biomarkers, protein biomarkers, and mRNA biomarkers [8]. Previous work from our laboratory showed differential mRNA expression in MetS in whole blood samples from the Arizona Insulin Resistance (AIR) registry [9]. In that study, we showed ninety altered circulating mRNAs with MetS, which could serve as potential biomarkers for the disease [9]. In more recent years, there has been a focus on identifying blood-based DNA methylation biomarkers for MetS.

Several studies showed an association of circulating DNA methylation biomarkers from either peripheral blood mononuclear cells (PBMCs), peripheral blood leukocytes (PBLs), or buffy coats with MetS [10–13]. These studies identified a number of DNA methylation biomarkers, including suppressor of cytokine signaling 3 (*SOCS3*) [11], ATP-binding cassette sub-family G member 1 (*ABCG1)* [10], Paternally Expressed 3 (*PEG*) [13], and Fatty Acid Binding Protein 3 (*FABP3*) [12]. Importantly, they linked the DNA methylation biomarker with the gene expression data. However, these aforementioned studies focused on Europeans and African Americans. Therefore, the role of whole blood DNA methylation biomarkers in MetS participants of Latino descent is less known and warrants investigation.

The present study aimed to measure whole blood DNA methylation in non-MetS *versus* MetS participants of Latino descent in the AIR registry. Specifically, we measured blood DNA methylation in three candidate genes: ATP Synthase, H+ Transporting mitochondrial F1 Complex, Epsilon Subunit (*ATP5E)*, Cytochrome C Oxidase Subunit VIc (*COX6C)*, and Ribosomal Protein L9 (*RPL9)*. We focused on *ATP5E*, *COX6C*, and *RPL9* because they were three of the top changing mRNAs (>1.5-fold change) in our previously published gene expression study of MetS participants in the AIR registry [9]. Moreover, these differentially expressed genes (*ATP5E*, *COX6C*, and *RPL9)* are part of the oxidative phosphorylation and ribosome pathways. We previously showed normalization of skeletal muscle protein levels for several ribosomal proteins in obese patients before and after bariatric surgery [14]. Moreover, we showed decreased mRNA expression for oxidative phosphorylation genes in skeletal muscle tissue of individuals with MetS characteristics [15, 16]. Therefore, these oxidative phosphorylation and ribosome genes/pathways are of interest to understand metabolic syndrome mechanisms.

For the present study, we measured promoter DNA methylation of *ATP5E*, *COX6C*, and *RPL9*. We focused on DNA methylation since this epigenetic modification can affect how genes are expressed [17–19]. We hypothesized that methylation would be lower in the promoters of *ATP5E*, *COX6C*, and *RPL9* in the MetS participants. Therefore, we hypothesized that there would be an inverse relationship between the previously published mRNA expression data from the AIR registry [9] with the methylation data for the genes of interest.

## Materials and methods

### Participants

All participants were of Latino descent from the Arizona Insulin Resistance (AIR) registry. Shaibi *et al*. previously described the goals of the biorepository [20]. Briefly, 667 participants took part in the AIR registry to establish a biobank for future investigation into diabetes and obesity-related health conditions in the Latino community. We selected a subset of the 667 participants for this present study (n = 184). We studied the same 184 participants that we had published global gene expression data for [9], in part, to look for an association with the methylation data. The 184 participants were selected randomly from the 667 participants. The only criteria used were age (≥18 years old). We realize that we may have inadvertently introduced a selection bias by not studying all the participants in the registry but a random subset aged 18 years or more.

We defined MetS as per the National Cholesterol Education Program Adult Treatment Panel III (NCEP: ATP III) [4]. Participants consented to the collection of anthropometric, demographic, family history, and medical history information. Measurements for weight (kg), body mass index (BMI) (kg/m^2^), waist circumference (cm) and seated blood pressure (mmHg) were collected. Following a 12-hour fast, participants agreed to blood samples to assess various metabolic phenotypes, including glucose, triglycerides, and high-density lipoprotein.

The Institutional Review Board (IRB) at Arizona State University approved the initial AIR registry study under protocol #0804002873. We obtained written consent from all participants. For the minors recruited into the AIR registry [although not studied as part of this project], we obtained written consent from their parents or guardians. Written consent was obtained to bank serum, DNA, and RNA and to use de-identified data and biospecimens for future studies, like the one described herein. The University of Arizona approved the present study under protocol #1703255156. The present study was considered exempt by the ethics committee at the University of Arizona since it utilized de-identified information of previously consented banked samples. Therefore, we made no recontact with these participants.

### Isolation of genomic DNA

Whole blood samples from the AIR registry participants were stored in PAXgene Blood Collection DNA Tubes (Qiagen Inc, Germantown, MD, USA) at -80°C until processed. We performed DNA extractions using the PAXgene Blood DNA Processing kit (Qiagen Inc, Germantown, MD, USA), as per the manufacturer’s instructions. The quality and quantity of the DNA were measured using a NanoDrop 2000 Spectrophotometer (ThermoFisher, Waltham, Massachusetts). The average DNA absorbance ratio (A260/A280) was 1.85 ± 0.05.

### Bisulfite conversion

As per the manufacturer’s instructions, the isolated genomic DNA was treated with bisulfite conversion using the EZ DNA Methylation-Lightning Kit (ZYMO, Irvine, CA, USA). The quantity of the bisulfite converted DNA was determined using the NanoDrop 2000 Spectrophotometer (Thermo Fisher Scientific, Waltham, MA, USA).

### Primer selection

Pyrosequencing primer sets were pre-designed and readily available for our candidate genes (*ATP5E*, *COX6C*, and *RPL9*) of interest (Qiagen Inc, Germantown, MD, USA). S1 Table provides the Qiagen catalog number, the target sequence to analyze, and the chromosomal location. The pre-designed primer sets were within the promoter of the gene of interest and were near the gene’s transcriptional start site. In addition, we selected these primer sets as the CpG coverage was between 4 to 6 per gene, which allowed us to perform CpG individual site methylation analysis and averaged analysis across the promoter of each gene. Lastly, we selected these pyrosequencing primer sets as computational PROMO analysis [21] revealed enrichment for potential transcription factors (S2 Table) that may bind in the target sequence of the candidate genes.

### PCR amplification and pyrosequencing

We added the bisulfite converted DNA (10 ng/μL) to the Qiagen PyroMark CpG assay PCR primer set along with the ZymoTaq Premix (ZYMO, Irvine, CA, USA). Samples were PCR amplified in a Veriti^®^ 96-Well Fast Thermal Cycler. We verified the quality of the PCR product with DNA Gel Electrophoresis using a Thermo Fisher Low Mass Ladder (ThermoFisher, Waltham, MA, USA). The amplified DNA was pyrosequenced on the Q48 Autoprep using the associated sequencing primer set from the PyroMark CpG assay kit (Qiagen Inc, Germantown, MD, USA), as per the manufacturer’s instructions. The PyroMark Q48 Advanced Software (Qiagen Inc, Germantown, MD, USA) calculates the percent of methylation. Specifically, it calculates methylation percentage from the ratio of the heights of a cytosine peak (methylated signal) and the sum of cytosine and thymine peaks (methylated and unmethylated signal) for each cytosine at the CpG site [22]. Methylated and unmethylated bisulfite converted DNA served as controls (Qiagen Inc, Germantown, MD, USA). In addition, we included non-template controls. Samples were performed in duplicate to confirm the reproducibility of methylation data.

### Statistical analysis

Statistical analyses were performed using STATA 14 (StataCorp, College Station, USA). Participant characteristic data was expressed as mean ± SEM, and categorical data were expressed as counts. The participant characteristic data was organized by MetS status, and statistical comparisons across the groups were performed using an independent sample t-test. The Shapiro-Wilk test was used to test for data normality. The pyrosequencing methylation data were not normally distributed. Therefore, the nonparametric Mann-Whitney U test was utilized. Categorical variables were analyzed using Pearson’s chi-square test. In addition, associations between MetS and DNA methylation were analyzed using logistic regression while controlling for age, gender, and smoking status in the model. The MetS group was the dependent variable with averaged gene methylation, age, gender (females used as the reference), and smoking status (nonsmokers used as the reference) as independent variables in the logistic regression analysis. Finally, Spearman’s correlation was performed to measure the strength and direction of the association between the methylation data and the previously published gene expression data. We used Partial Spearman’s Rank Correlation to adjust for age, gender, and smoking status for the methylation and gene expression correlation. Significance was set at p < 0.05 for all tests.

## Results

### Participants

Of the 184 participants in the present study, 74 met the criteria for MetS. The remaining 110 participants classified as non-MetS. As expected, MetS participants had significantly higher blood pressure measures, waist circumference, triglycerides and fasting plasma glucose. Moreover, the MetS group showed lower high-density lipoprotein levels (Table 1). However, there was no significant gender or smoking status difference between the non-MetS versus MetS (Table 1).

**Table 1:** Phenotypes from non-MetS *versus* MetS participants in the Arizona Insulin Resistance (AIR) registry.

Phenotype	Non-MetS	MetS	P Value
Gender	36M/74F	25M/49F	NS
Age	35 ± 1	39 ± 1	<0.01
Systolic Blood Pressure (mmHg)	114.7 ± 1.1	128.4 ± 2.4	<0.0001
Diastolic Blood Pressure (mmHg)	75.3 ± 0.8	81.5 ± 1.2	<0.0001
Waist Circumference (cm)	95.1 ± 1.2	109.1 ± 1.7	<0.0001
Triglycerides (mg/dL)	107.7 ± 4.3	204.7 ± 12.2	<0.0001
Fasting Plasma Glucose (mg/dL)	92.5 ± 1.8	112.8 ± 4.8	<0.0001
High Density Lipoprotein (mg/dL)	47.8 ± 1.1	37.6 ± 1.0	<0.0001
Smoking status	91 No/18 Yes/1 UNK	63 No/11 Yes	NS

Data are means ± SEM. UNK = unknown.

### Differential DNA methylation between MetS and non-MetS groups

**Fig 1:**
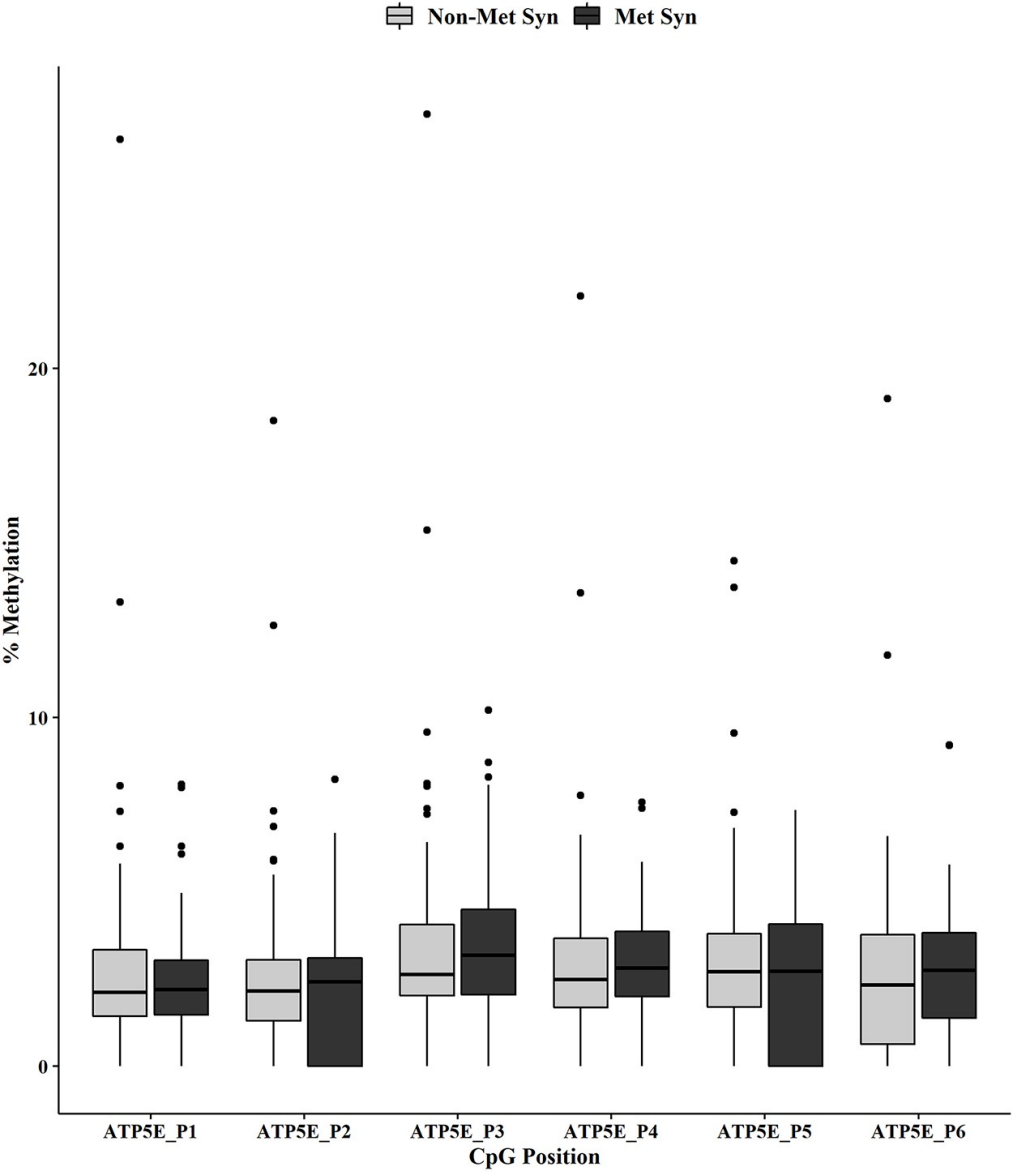
*ATP5E* promoter DNA methylation by MetS status. DNA methylation at six CpGs within the promoter of *ATP5E* was measured in non-MetS (n = 110) and MetS (n = 74). Mann-Whitney U test was used for the statistical comparisons between the non-MetS *versus* MetS.

**Fig 2:**
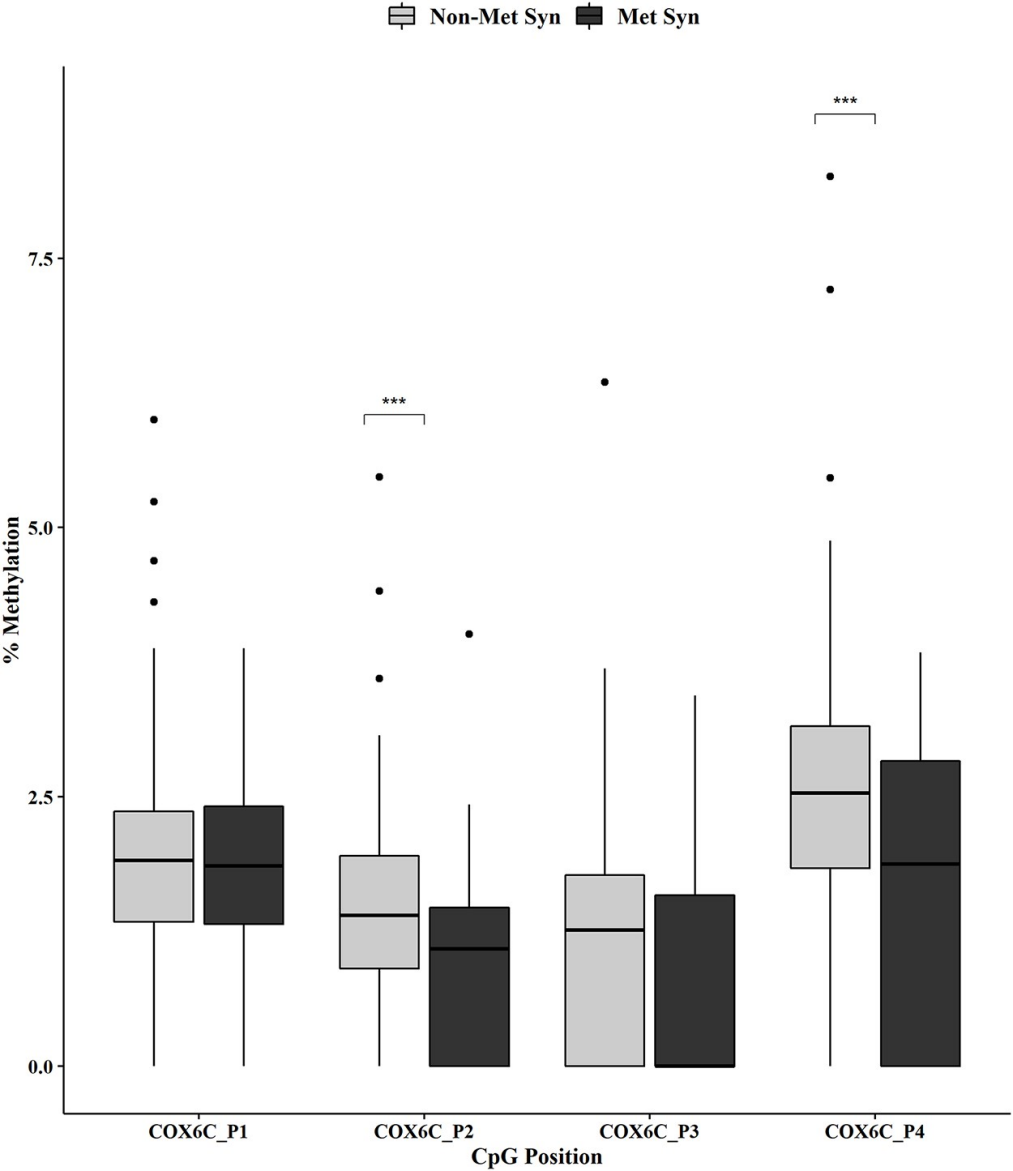
*COX6C* promoter DNA methylation by MetS status. DNA methylation at four CpGs within the promoter of *COX6C* was measured in non-MetS (n = 110) and MetS (n = 74). Mann-Whitney U test was used for the statistical comparisons between the non-MetS *versus* MetS. ***p < 0.001.

**Fig 3:**
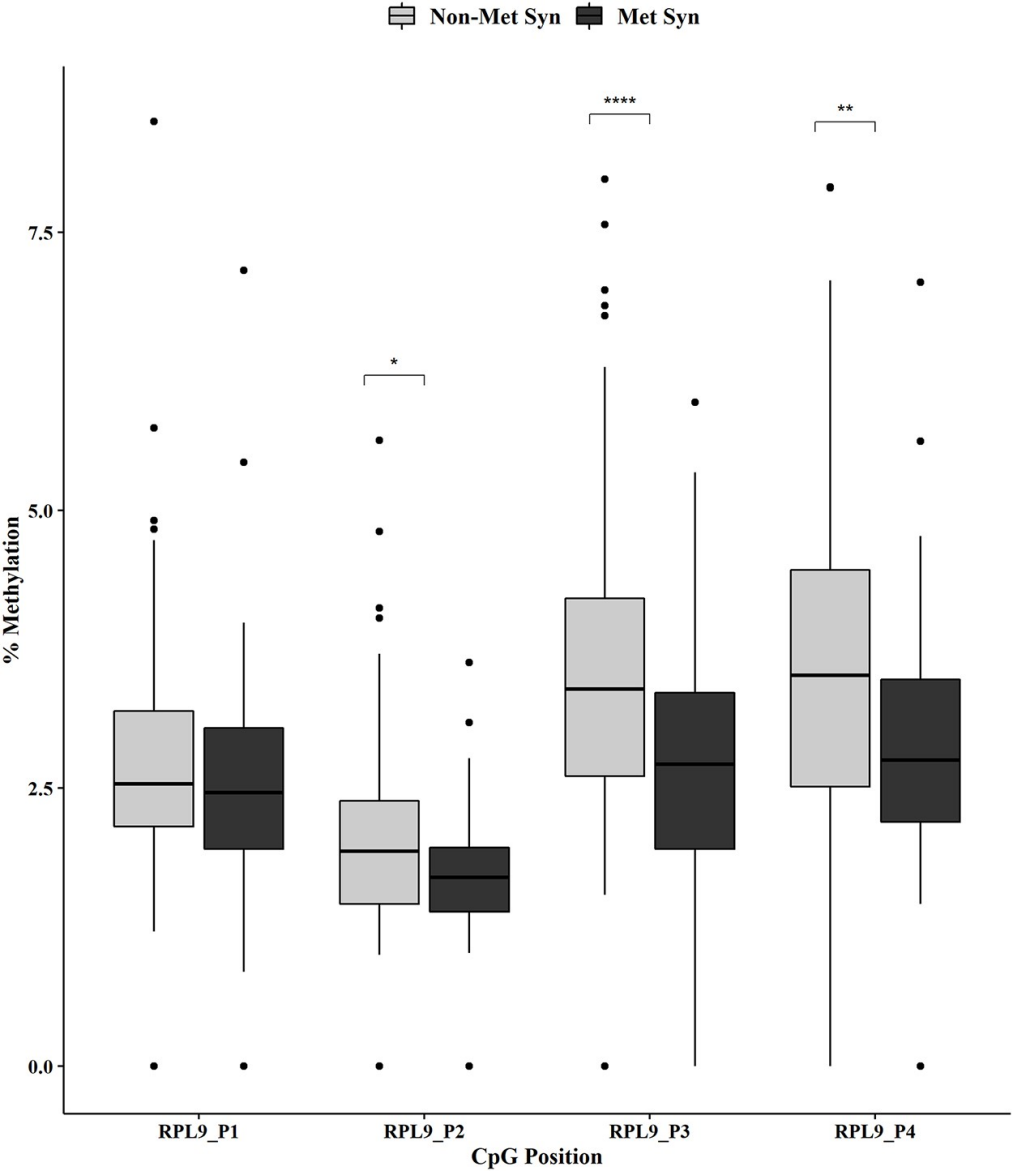
*RPL9* promoter DNA methylation by MetS status. DNA methylation at four CpGs within the promoter of *RPL9* was measured in non-MetS (n = 110) and MetS (n = 74). Mann-Whitney U test was used for the statistical comparisons between the non-MetS *versus* MetS. *p < 0.05, **p < 0.01, ****p < 0.0001.

Individual CpG methylation positions within each gene were analyzed by MetS status using the Mann-Whitney U test. We showed no change in methylation for the *ATP5E* CpG positions across the non-MetS *versus* MetS groups (Fig 1). However, we did observe a significant decrease in methylation for *COX6C* at CpG positions 2 and 4 in the MetS group (Fig 2, both p < 0.001). In addition, the *RPL9* methylation analysis revealed a significant decreased methylation for positions 2 (p < 0.05), 3 (p < 0.0001) and 4 (p < 0.01) in the MetS group (Fig 3).

**Fig 4:**
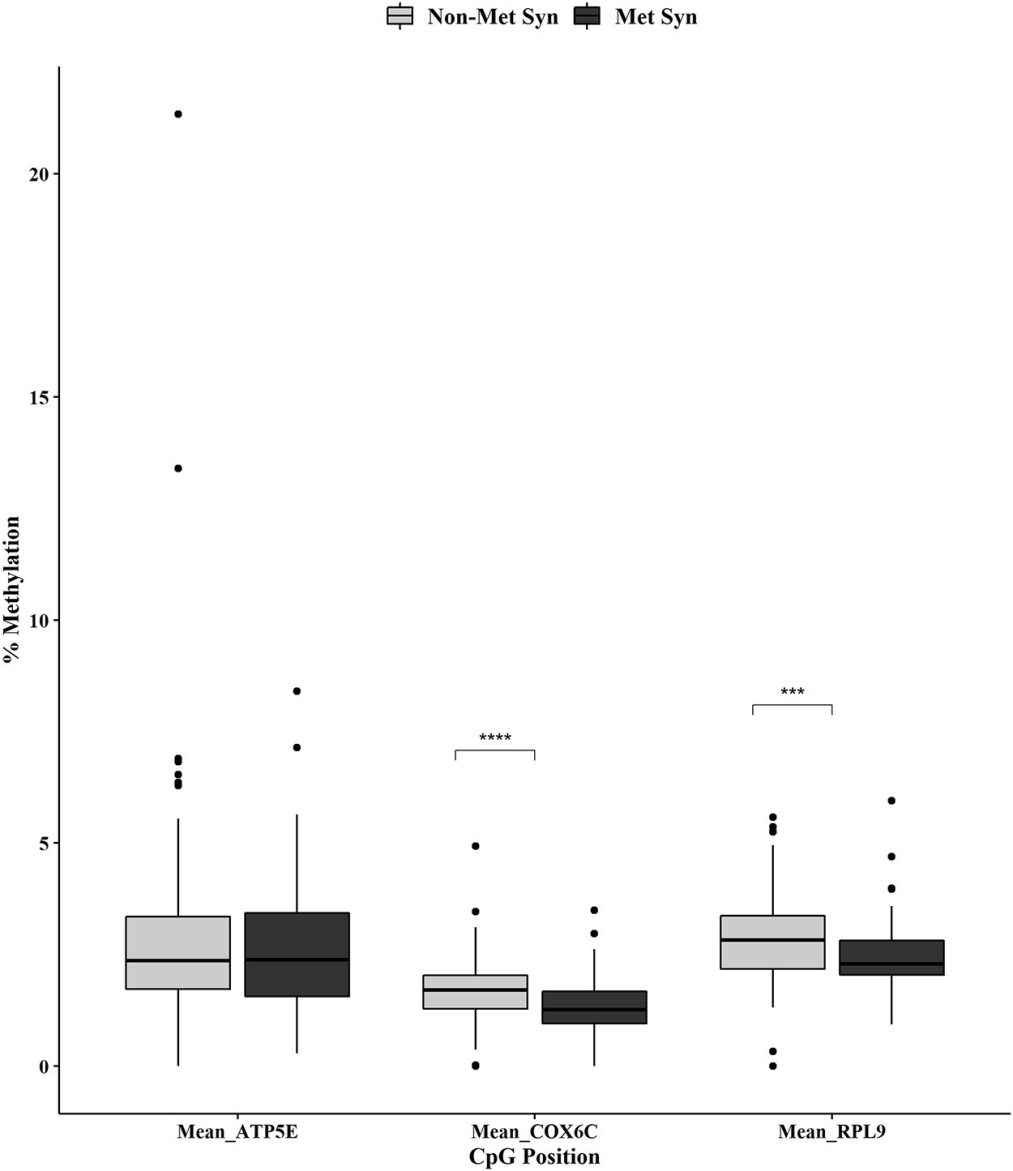
*ATP5E*, *COX6C* and *RPL9* overall promoter DNA methylation by MetS status. Mean methylation within the promoters of *ATP5E*, *COX6C* and *RPL9* in non-MetS (n = 110) and MetS (n = 74). Mann-Whitney U test was used for the statistical comparisons between the non-MetS *versus* MetS. ***p < 0.001, ****p < 0.0001.

We used the Mann-Whitney U test to determine mean methylation differences when averaging across the CpG positions for *ATP5E*, *COX6C*, and *RPL9*. The averaged methylation within the promoter of *ATP5E* was not significantly different between the non-MetS and MetS groups (Fig 4). However, the averaged methylation across the promoters for *COX6C* and *RPL9* were both significantly decreased (p < 0.0001 and p < 0.001, respectively, Fig 4).

### Association of promoter methylation with MetS status

Logistic regression analysis assessed whether averaged promoter methylation for *ATP5E*, *COX6C*, and *RPL9* was associated with MetS when controlling for potential confounding factors such as age, gender, and smoking status. As expected, we observed no association for the mean *ATP5E* methylation with MetS. However, mean methylation for the promoter of *COX6C* was significantly associated with MetS when controlling for age, gender, and smoking status [the adjusted odds ratio: 0.36, 95% confidence interval: 0.21–0.60], p < 0.001. Similarly, the mean methylation for the promoter of *RPL9* was significantly associated with MetS when controlling for age, gender, and smoking status [the adjusted odds ratio: 0.56, 95% confidence interval: 0.38–0.82], p < 0.01.

### Correlation analysis of gene expression data with methylation data

**Fig 5:**
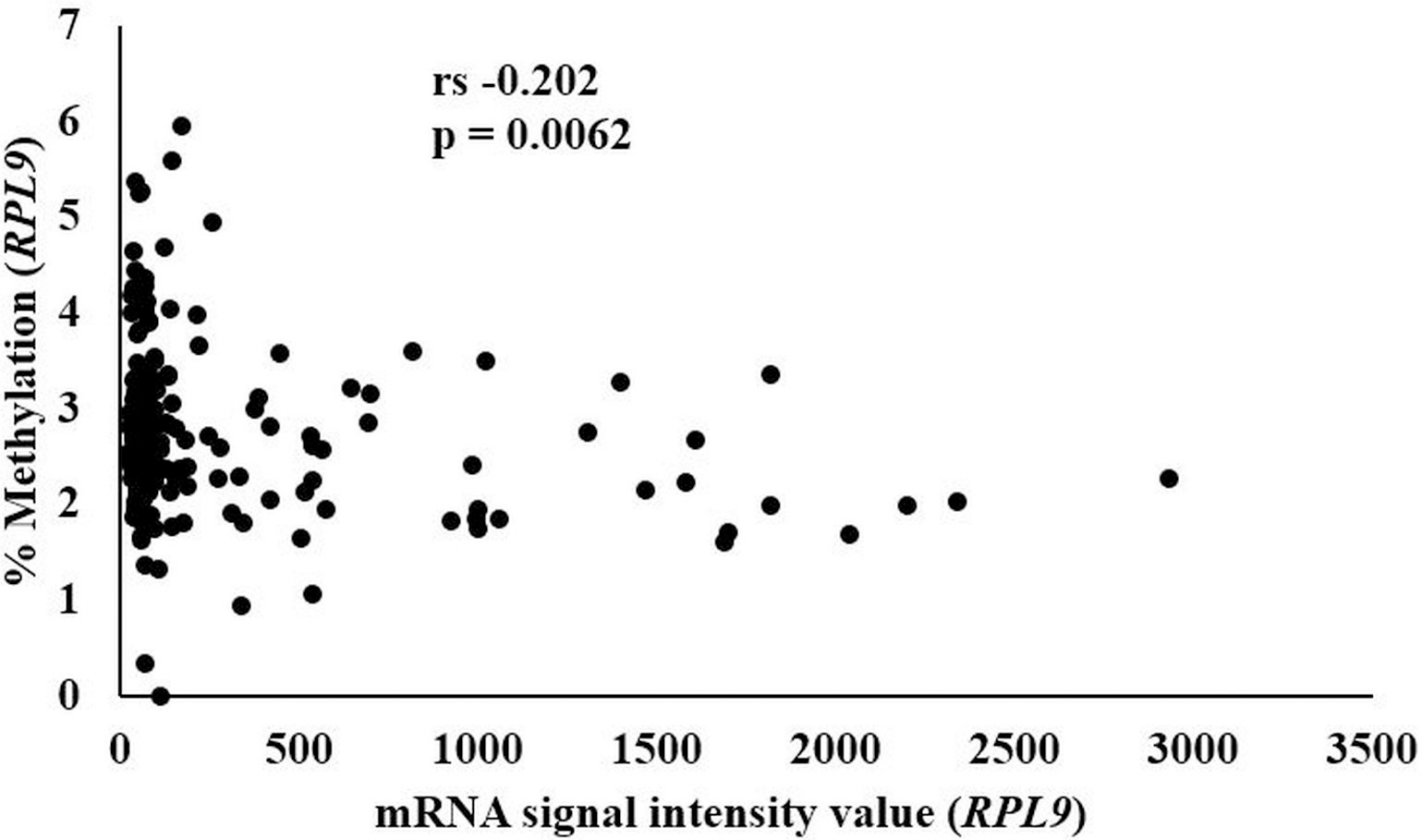
Correlation analysis between *RPL9* mRNA and *RPL9* methylation. Spearman’s correlation was used to calculate the strength and direction of the relationship between the *RPL9* mRNA and *RPL9* methylation data. Rho (rs) value of rs -0.202, p = 0.0062.

We performed a Spearman’s correlation between the previously published gene expression data [9] with the averaged methylation for *ATP5E*, *COX6C*, and *RPL9*. *ATP5E* gene expression was not correlated to the mean methylation as demonstrated with a Rho (rs) value of 0.117, p = NS. Likewise, the gene expression data for *COX6C* did not correlate to the mean methylation (rs -0.088, p = NS). However, the averaged methylation for *RPL9* showed a significant inverse correlation with the previously published gene expression data (rs -0.202, p = 0.0062, Fig 5). When we adjusted for age, sex, and smoking status using the Partial Spearman’s Rank Correlation, the significance was p = 0.0030.

## Discussion

This study set out to identify potential DNA methylation biomarkers in whole blood samples from participants of Latino descent from the AIR registry. In particular, we focused on select genes (*ATP5E*, *COX6C*, and *RPL9)* in non-MetS *versus* MetS participants. We focused on these genes since we previously showed altered gene expression of *ATP5E*, *COX6C*, and *RPL9* in MetS participants from the AIR registry [9]. In that study, we demonstrated an increased gene expression for *ATP5E*, *COX6C*, and *RPL9* in the MetS group [9]. In the present study, we showed a decreased methylation of *COX6C* and *RPL9* in participants with MetS while controlling for potential confounding variables such as age, gender, and smoking status. We found no association of *ATP5E* DNA methylation with MetS in the AIR registry samples. Our findings provide evidence for reduced methylation of cytosines in the promoter region of our select candidate genes, specifically *COX6C* and *RPL9*. Taken collectively, *COX6C* and *RPL9* may be useful as potential blood-based DNA methylation biomarkers for MetS.

ATP Synthase, H+ Transporting mitochondrial F1 Complex, Epsilon Subunit (*ATP5E*) was the first gene-targeted in this blood-based DNA methylation biomarker study. *ATP5E* is a subunit of the mitochondrial ATP synthase and is essential for oxidative phosphorylation. We previously showed a collection of whole blood oxidative phosphorylation genes that were increased in the MetS participants [9]. Furthermore, in type 2 diabetic mouse liver models, the oxidative phosphorylation gene *ATP5E* was upregulated in the diabetic livers compared to control livers [23]. Based on these aforementioned studies, we pursued this gene as a blood-based DNA methylation biomarker. We showed no difference in methylation between the non-MetS group *versus* the MetS group in the *ATP5E* gene. In this study, we focused on 6 CpG sites within the promoter of *ATP5E*. Based on these findings, we suggest that we did not target enough CpG sites within the *ATP5E*. Moreover, the target sequence we measured was likely not important for gene regulation.

Another oxidative phosphorylation gene that we targeted was cytochrome C oxidase subunit 6c (*COX6C*). *COX6C* is a subunit of the Complex IV protein structure in the mitochondrial respiratory chain [24], and it regulates the formation or activity of Complex IV in the electron transport chain [25]. Many studies have implicated a role for *COX6C* in metabolic diseases. For example, a microarray study of the aorta and adipose tissue showed a decreased expression for COX6C in insulin-resistant and atherosclerotic obese mouse models [26]. Moreover, an analysis of type 2 diabetic Goto-Kakizaki mice revealed a differential gene expression of *COX6C* in liver samples [27]. The present study’s findings supported our overall hypothesis, wherein we observed a decreased methylation in *COX6C* in whole blood in the AIR registry MetS participants. However, we found no significant correlation when we performed Spearman’s correlation between the methylation data with the previously published gene expression data [9]. The lack of association with the mRNA data suggests that the methylation sites we measured were not important for gene regulation.

The human ribosomal protein L9 (*RPL9*) gene codes for a subunit of the 60S subunit or the large ribosomal subunit [28]. Ribosomal proteins, like *RPL9*, serve to stabilize specific rRNAs by binding directly and assist in ensuring correct structural alterations to rRNA during ribosome assembly [29]. We chose to study the *RPL9* gene for multiple reasons. Our laboratory showed a role for ribosomal proteins in obese patients before and after bariatric surgery [14]. In addition, there have been many studies implicating ribosomal genes/proteins in metabolic diseases. For example, a transcriptomic analysis of samples from non-steatotic and steatotic livers of severely obese adolescents showed a significant downregulation for *RPL9* in the steatotic population [30]. In addition, a study in liver mitochondrial fractions showed an increase of >1.5-fold in *RPL9* in those mice fed a western diet *versus* a standard diet [31]. In the present study, we showed significant changes in DNA methylation in the MetS participants. In particular, we showed that the methylation was decreased significantly in 3 out of 4 of the CpG sites captured.

We hypothesized that there would be a significant inverse relationship between the previously published gene expression data [9] with the methylation data for the genes of interest. Spearman’s correlation showed that averaged methylation across the *RPL9* promoter was significantly associated with the previously published gene expression data. The *RPL9* findings support our hypothesis that the reduction of methylation in the promoter region would correlate with the previously observed increased *RPL9* gene expression data [9]. Although we did not directly measure it, we can hypothesize there is an increase in the cellular content of ribosomes in the blood of MetS participants. The increase in cellular content of ribosomes may increase protein synthesis to account for the increased energy demands observed in MetS participants [32]. A study by Hoppe *et al*. showed a direct correlation between energy demands and ribosomal RNA synthesis [33]. Their findings suggest that ribosomal RNA synthesis increases when mitogenic conditions are optimal, for example, an overabundance of available nutrients. Taken together, *RPL9* is a promising candidate gene, and future studies are warranted for understanding the exact role of ribosomal proteins in whole blood and skeletal muscle in metabolic diseases.

Some limitations apply to this study that warrants a discussion in the context of our findings. Firstly, we did not experimentally measure whether the *RPL9* methylation sites directly alter gene expression. Future studies are warranted to address this wherein we would perform an *in vitro* methylation luciferase assay to measure the effect of methylation in the promoters of the select genes (*ATP5E*, *COX6C*, and *RPL9*) on gene expression levels [34]. Secondly, another limitation is the target sequences presented in this study captured only 4–6 CpG sites for each gene studied. In comparison, another study analyzed over 20 CpG sites for one target gene [12]. Measuring more CpGs would provide more insight into the epigenetic landscape of the promoter of these target genes on the MetS phenotype. A future study would be to design additional CpG assays covering the promoter region for each gene. Thirdly, our laboratory studies metabolic disease in whole blood and skeletal muscle, and we have previously shown contradictory findings between these two tissues [9]. This study was unable to measure skeletal muscle methylation of these candidate genes. A future study would be to perform methylation analysis of *ATP5E*, *COX6C*, and *RPL9* in skeletal muscle of MetS and non-MetS participants. Fourthly, we did not validate our findings in an independent cohort nor other populations. Validating these DNA methylation biomarkers in larger studies would be critical for determining if they would be suitable to function as biomarkers, and ultimately be utilized in clinical practice for screening and diagnosing MetS. Lastly, another limitation of this study is the random selection of the 184 participants used for the previously published gene expression study [9] and the present study. The age criteria may have introduced a bias into the analysis.

In conclusion, we showed decreased whole blood DNA methylation for *COX6C* and *RPL9* in participants with MetS. Moreover, we provide evidence of a significant inverse relationship between the gene expression and methylation data for *RPL9*. Our findings contribute to the field of whole blood-based DNA methylation biomarkers [10–13], specifically highlighting MetS biomarkers in Latino participants. Our novel potential epigenetic biomarkers should be measured in other populations and importantly validated in larger cohorts.

## Supporting information

S1 Table Pyrosequencing primer set information. (PDF)

S2 Table PROMO transcription factor analysis of pyrosequencing target gene regions. (PDF)

## References

[1] Samson SL, Garber AJ. Metabolic syndrome. Endocrinol Metab Clin North Am. 2014;43(1):1–23. Epub 2014/03/04. doi: 10.1016/j.ecl.2013.09.009 . 24582089

[2] Zhang X, Cui X, Li F, Wang S, Liu X, Hui L, et al. Association between diabetes mellitus with metabolic syndrome and diabetic microangiopathy. Exp Ther Med. 2014;8(6):1867–73. Epub 2014/11/06. doi: 10.3892/etm.2014.1992 25371746 PMC4217776

[3] Liu L, Miura K, Fujiyoshi A, Kadota A, Miyagawa N, Nakamura Y, et al. Impact of metabolic syndrome on the risk of cardiovascular disease mortality in the United States and in Japan. Am J Cardiol. 2014;113(1):84–9. Epub 2013/10/31. doi: 10.1016/j.amjcard.2013.08.042 . 24169008

[4] National Cholesterol Education Program Expert Panel on Detection E, Treatment of High Blood Cholesterol in A. Third Report of the National Cholesterol Education Program (NCEP) Expert Panel on Detection, Evaluation, and Treatment of High Blood Cholesterol in Adults (Adult Treatment Panel III) final report. Circulation. 2002;106(25):3143–421. Epub 2002/12/18. . 12485966

[5] Moore JX, Chaudhary N, Akinyemiju T. Metabolic Syndrome Prevalence by Race/Ethnicity and Sex in the United States, National Health and Nutrition Examination Survey, 1988–2012. Prev Chronic Dis. 2017;14:E24. Epub 2017/03/17. doi: 10.5888/pcd14.160287 28301314 PMC5364735

[6] Rochlani Y, Pothineni NV, Kovelamudi S, Mehta JL. Metabolic syndrome: pathophysiology, management, and modulation by natural compounds. Ther Adv Cardiovasc Dis. 2017;11(8):215–25. Epub 2017/06/24. doi: 10.1177/1753944717711379 28639538 PMC5933580

[7] Srikanthan K, Feyh A, Visweshwar H, Shapiro JI, Sodhi K. Systematic Review of Metabolic Syndrome Biomarkers: A Panel for Early Detection, Management, and Risk Stratification in the West Virginian Population. Int J Med Sci. 2016;13(1):25–38. Epub 2016/01/28. doi: 10.7150/ijms.13800 26816492 PMC4716817

[8] O’Neill S, Bohl M, Gregersen S, Hermansen K, O’Driscoll L. Blood-Based Biomarkers for Metabolic Syndrome. Trends Endocrinol Metab. 2016;27(6):363–74. Epub 2016/05/07. doi: 10.1016/j.tem.2016.03.012 . 27150849

[9] Tangen SE, Tsinajinnie D, Nunez M, Shaibi GQ, Mandarino LJ, Coletta DK. Whole blood gene expression profiles in insulin resistant Latinos with the metabolic syndrome. PLoS One. 2013;8(12):e84002. Epub 2013/12/21. doi: 10.1371/journal.pone.0084002 24358323 PMC3866261

[10] Akinyemiju T, Do AN, Patki A, Aslibekyan S, Zhi D, Hidalgo B, et al. Epigenome-wide association study of metabolic syndrome in African-American adults. Clin Epigenetics. 2018;10:49. Epub 2018/04/13. doi: 10.1186/s13148-018-0483-2 neutral with regard to jurisdictional claims in published maps and institutional affiliations. 29643945 PMC5891946

[11] Ali O, Cerjak D, Kent JW Jr., James R, Blangero J, Carless MA, et al. Methylation of SOCS3 is inversely associated with metabolic syndrome in an epigenome-wide association study of obesity. Epigenetics. 2016;11(9):699–707. Epub 2016/08/27. doi: 10.1080/15592294.2016.1216284 27564309 PMC5048720

[12] Zhang Y, Kent JW 2nd, Lee A, Cerjak D, Ali O, Diasio R, et al. Fatty acid binding protein 3 (fabp3) is associated with insulin, lipids and cardiovascular phenotypes of the metabolic syndrome through epigenetic modifications in a Northern European family population. BMC Med Genomics. 2013;6:9. Epub 2013/03/21. doi: 10.1186/1755-8794-6-9 23510163 PMC3608249

[13] van Otterdijk SD, Binder AM, Szarc Vel Szic K, Schwald J, Michels KB. DNA methylation of candidate genes in peripheral blood from patients with type 2 diabetes or the metabolic syndrome. PLoS One. 2017;12(7):e0180955. Epub 2017/07/21. doi: 10.1371/journal.pone.0180955 28727822 PMC5519053

[14] Campbell LE, Langlais PR, Day SE, Coletta RL, Benjamin TR, De Filippis EA, et al. Identification of Novel Changes in Human Skeletal Muscle Proteome After Roux-en-Y Gastric Bypass Surgery. Diabetes. 2016;65(9):2724–31. Epub 2016/05/22. doi: 10.2337/db16-0004 27207528 PMC5001187

[15] Coletta DK, Mandarino LJ. Mitochondrial dysfunction and insulin resistance from the outside in: extracellular matrix, the cytoskeleton, and mitochondria. Am J Physiol Endocrinol Metab. 2011;301(5):E749–55. Epub 2011/08/25. doi: 10.1152/ajpendo.00363.2011 21862724 PMC3214002

[16] Patti ME, Butte AJ, Crunkhorn S, Cusi K, Berria R, Kashyap S, et al. Coordinated reduction of genes of oxidative metabolism in humans with insulin resistance and diabetes: Potential role of PGC1 and NRF1. Proc Natl Acad Sci U S A. 2003;100(14):8466–71. Epub 2003/07/02. doi: 10.1073/pnas.1032913100 12832613 PMC166252

[17] Noble D. Conrad Waddington and the origin of epigenetics. J Exp Biol. 2015;218(Pt 6):816–8. Epub 2015/03/20. doi: 10.1242/jeb.120071 . 25788723

[18] Russo VEA, Martienssen RA, Riggs AD. Epigenetic mechanisms of gene regulation. Plainview, N.Y.: Cold Spring Harbor Laboratory Press; 1996. xii, 692 p. p.

[19] Moore LD, Le T, Fan G. DNA methylation and its basic function. Neuropsychopharmacology. 2013;38(1):23–38. Epub 2012/07/12. doi: 10.1038/npp.2012.112 22781841 PMC3521964

[20] Shaibi GQ, Coletta DK, Vital V, Mandarino LJ. The design and conduct of a community-based registry and biorepository: a focus on cardiometabolic health in Latinos. Clin Transl Sci. 2013;6(6):429–34. Epub 2013/10/15. doi: 10.1111/cts.12114 24119012 PMC4225082

[21] Messeguer X, Escudero R, Farre D, Nunez O, Martinez J, Alba MM. PROMO: detection of known transcription regulatory elements using species-tailored searches. Bioinformatics. 2002;18(2):333–4. Epub 2002/02/16. doi: 10.1093/bioinformatics/18.2.333 . 11847087

[22] Sestakova S, Salek C, Remesova H. DNA Methylation Validation Methods: a Coherent Review with Practical Comparison. Biol Proced Online. 2019;21:19. Epub 2019/10/05. doi: 10.1186/s12575-019-0107-z 31582911 PMC6771119

[23] Zhang F, Xu X, Zhang Y, Zhou B, He Z, Zhai Q. Gene expression profile analysis of type 2 diabetic mouse liver. PLoS One. 2013;8(3):e57766. Epub 2013/03/08. doi: 10.1371/journal.pone.0057766 23469233 PMC3585940

[24] Schweppe DK, Chavez JD, Lee CF, Caudal A, Kruse SE, Stuppard R, et al. Mitochondrial protein interactome elucidated by chemical cross-linking mass spectrometry. Proc Natl Acad Sci U S A. 2017;114(7):1732–7. Epub 2017/01/29. doi: 10.1073/pnas.1617220114 28130547 PMC5321032

[25] Pierron D, Wildman DE, Huttemann M, Markondapatnaikuni GC, Aras S, Grossman LI. Cytochrome c oxidase: evolution of control via nuclear subunit addition. Biochim Biophys Acta. 2012;1817(4):590–7. Epub 2011/08/02. doi: 10.1016/j.bbabio.2011.07.007 21802404 PMC3923406

[26] Moreno-Viedma V, Amor M, Sarabi A, Bilban M, Staffler G, Zeyda M, et al. Common dysregulated pathways in obese adipose tissue and atherosclerosis. Cardiovasc Diabetol. 2016;15(1):120. Epub 2016/08/27. doi: 10.1186/s12933-016-0441-2 27561966 PMC5000404

[27] Deng WJ, Nie S, Dai J, Wu JR, Zeng R. Proteome, phosphoproteome, and hydroxyproteome of liver mitochondria in diabetic rats at early pathogenic stages. Mol Cell Proteomics. 2010;9(1):100–16. Epub 2009/08/25. doi: 10.1074/mcp.M900020-MCP200 19700791 PMC2808256

[28] Mazuruk K, Schoen TJ, Chader GJ, Iwata T, Rodriguez IR. Structural organization and chromosomal localization of the human ribosomal protein L9 gene. Biochim Biophys Acta. 1996;1305(3):151–62. Epub 1996/03/01. doi: 10.1016/0167-4781(95)00201-4 . 8597601

[29] Baik IH, Jo GH, Seo D, Ko MJ, Cho CH, Lee MG, et al. Knockdown of RPL9 expression inhibits colorectal carcinoma growth via the inactivation of Id-1/NF-kappaB signaling axis. Int J Oncol. 2016;49(5):1953–62. Epub 2016/10/26. doi: 10.3892/ijo.2016.3688 . 27633352

[30] Sheldon RD, Kanosky KM, Wells KD, Miles L, Perfield JW 2nd, Xanthakos S, et al. Transcriptomic differences in intra-abdominal adipose tissue in extremely obese adolescents with different stages of NAFLD. Physiol Genomics. 2016;48(12):897–911. Epub 2016/10/21. doi: 10.1152/physiolgenomics.00020.2016 27764764 PMC5206389

[31] Einer C, Hohenester S, Wimmer R, Wottke L, Artmann R, Schulz S, et al. Data on chow, liver tissue and mitochondrial fatty acid compositions as well as mitochondrial proteome changes after feeding mice a western diet for 6–24 weeks. Data Brief. 2017;15:163–9. Epub 2017/10/17. doi: 10.1016/j.dib.2017.09.019 29034285 PMC5633826

[32] Ghosh S, Dent R, Harper ME, Gorman SA, Stuart JS, McPherson R. Gene expression profiling in whole blood identifies distinct biological pathways associated with obesity. BMC Med Genomics. 2010;3:56. Epub 2010/12/03. doi: 10.1186/1755-8794-3-56 21122113 PMC3014865

[33] Hoppe S, Bierhoff H, Cado I, Weber A, Tiebe M, Grummt I, et al. AMP-activated protein kinase adapts rRNA synthesis to cellular energy supply. Proc Natl Acad Sci U S A. 2009;106(42):17781–6. Epub 2009/10/10. doi: 10.1073/pnas.0909873106 19815529 PMC2764937

[34] Han W, Shi M, Spivack SD. Site-specific methylated reporter constructs for functional analysis of DNA methylation. Epigenetics. 2013;8(11):1176–87. Epub 2013/09/06. doi: 10.4161/epi.26195 . 24004978

