# Supplementary material for: Potential evidence for epigenetic biomarkers of metabolic syndrome in human whole blood in Latinos
Source: PLoS One. 2021 Oct 29;16(10):e0259449. doi: 10.1371/journal.pone.0259449 (PMC8555810; doi:10.1371/journal.pone.0259449)
Supplement: S1 Table — (PDF) [file pone.0259449.s001.pdf]

S1 Table. Primer set information

Genes of Interest	Qiagen Catalog Number	Chromosomal Region[a]	Target Sequence[b]
ATP5E	PM00018172	Chr20:57,607,314 – 57,607,340	CACCATGCTGTAG**C**GAAAG**C**GGAGCT**C**GT**C**GGGC**C**GAAT**C**GC
COX6C	PM00137830	Chr8:100,905,758 – 100,905,787	A**C**GG**C**GGAGACACACAGTCA**C**GACTAAATC**C**GA
RPL9	PM00196392	Chr4:39,460,177 – 39,460,198	ACACTGGGGCCC**G**CTGTC**G**GTCTCCC**G**TCCTCC**G**A

[a]Chromosomal regions based on GRCh37 Genome Reference Consortium Human Build 37 (GRCh37)

[b]The underlined C or G indicates the position of the CpG sites in the genes of interest
