# Supplementary material for: Potential evidence for epigenetic biomarkers of metabolic syndrome in human whole blood in Latinos
Source: PLoS One. 2021 Oct 29;16(10):e0259449. doi: 10.1371/journal.pone.0259449 (PMC8555810; doi:10.1371/journal.pone.0259449)
Supplement: S2 Table — (PDF) [file pone.0259449.s002.pdf]

S2 Table. PROMO transcription factor analysis of pyrosequencing target gene regions

Gene	Target Sequence for PROMO analysis	Putative Transcription Factor Binding Sites[a]
ATP5E	CACCATGCTGTAG**C**GAAAG**C**GGAGCT**C**GT**C**GGGC**C**GAAT**C**GC	YY1, XBP-1, GR-beta, Pax-5, p53, E2F-1
COX6C	A**C**GG**C**GGAGACACACAGTCA**C**GACTAAATC**C**GA	GR-beta, c-Jun, TFII-I, E2F-1, TFII-I, HNF-3alpha, AP-1
RPL9	ACACTGGGGCCC**G**CTGTC**G**GTCTCCC**G**TCCTCC**G**A	Pax-5, p53, E2F-1, TFII-I, GR-alpha, PPAR-alpha: RXR-alpha

[a]Factors predicted within a dissimilarity margin les or equal than 15%
